# Elastic Stable Intramedullary Nailing (ESIN), Orthoss^® ^and Gravitational Platelet Separation - System (GPS^®^): An effective method of treatment for pathologic fractures of bone cysts in children

**DOI:** 10.1186/1471-2474-12-45

**Published:** 2011-02-12

**Authors:** Marion Rapp, Daniel Svoboda, Lucas M Wessel, Martin M Kaiser

**Affiliations:** 1 Department of Pediatric Surgery, University of Luebeck, Luebeck, Germany; 2 Department of Pediatric Surgery, University of Heidelberg, Mannheim, Germany

## Abstract

**Background:**

The different treatment strategies for bone cysts in children are often associated with persistence and high recurrence rates of the lesions. The safety and clinical outcomes of a combined mechanical and biological treatment with elastic intramedullary nailing, artificial bone substitute and autologous platelet rich plasma are evaluated.

**Methods:**

From 02/07 to 01/09 we offered all children with bone cysts the treatment combination of elastic intramedullary nailing (ESIN), artificial bone substitute (Orthoss^®^) and autologous platelet rich plasma, concentrated by the Gravitational Platelet Separation (GPS^®^) - System. All patients were reviewed radiologically for one year following the removal of the intramedullary nailing, which was possible because of cyst obliteration.

**Results:**

A cohort of 12 children (4 girls, 8 boys) was recruited. The mean patient age was 11.4 years (range 7-15 years). The bone defects (ten humeral, two femoral) included eight juvenile and four aneurysmal bone cysts. Five patients suffered from persistent cysts following earlier unsuccessful treatment of humeral bone cyst after pathologic fracture; the other seven presented with acute pathologic fractures. No peri- or postoperative complications occurred. The radiographic findings showed a total resolution of the cysts in ten cases (Capanna Grade 1); in two cases a small residual cyst remained (Capanna Grade 2). The intramedullary nails were removed six to twelve months (mean 7.7) after the operation; in one case, a fourteen year old boy (Capanna Grade 2), required a further application of GPS^® ^and Orthoss^® ^to reach a total resolution of the cyst. At follow-up (20-41 months, mean 31.8 months) all patients showed very good functional results and had returned to sporting activity. No refracture occurred, no further procedure was necessary.

**Conclusions:**

The combination of elastic intramedullary nailing, artificial bone substitute and autologous platelet rich plasma (GPS^®^) enhances the treatment of bone cysts in children, with no resulting complications.

## Background

Juvenile bone cysts were first described by Virchow in 1876, but their aetiology still remains unknown [1]. They can occur in any bone, most often in the long bones and at any age, but mainly in the first two decades [2]. Despite their benign nature, simple bone cysts interfere with everyday activities. This is because the cysts weaken the cortex, predisposing the bone to pathological fracture. Various treatment options have been reported apart from the principle of 'watch and wait' for spontaneous consolidation. The filling of the cysts with cortisone [3-6], bone marrow [7-9] or allogenic bone grafts [10-12] have been described. Another approach is to stabilize the cyst with elastic stable intramedullary nails, allowing for immediate mobilization. This procedure can also be combined with a bone substitute [13,14] or the decompression of the cyst with cannulated screws [15]. However, none of these treatments has been evaluated as being superior to the others in terms of avoiding the persistence of the condition, recurrence or refractures [2,16].

In the case of a fracture, a healing process is initiated with fibrin clot formation, platelet aggregation and degranulation. Platelets contain numerous growth factors such as platelet-derived growth factor, transforming growth factor beta, insulin-like growth factors I and II and epidermal growth factor [17-19]. Experimental studies have shown that a gravitational platelet-separating system is able to boost the concentration of growth factors. This has the potential to stimulate the prematurely terminated bone-healing processes, especially when combined with autologous bone or bone graft materials [20-22]. Initial published studies on the use of autologous concentrated platelets in poorly healing dermal wounds [23,24] and in artificial joint surgery [25] have also demonstrated its efficiency.

Nowadays in our dynamic and sportive society, children and adolescents want to return to all activities as soon as possible and are afraid of refractures of the cysts arising from minor trauma. Driven by our own mediocre results during the treatment of juvenile bone cysts with prednisolone, cannulated "decompression" screws or ESIN in isolation, we looked for an alternative additional treatment strategy to hasten healing and to minimize the need for repeat operations. This study evaluates the safety and clinical outcome of the treatment with elastic intramedullary nailing (ESIN), artificial bone substitute (Orthoss^®^) and autologous platelet rich plasma (GPS^®^) in bone cysts in children.

## Methods

From February 2007 to January 2009 we offered a combined treatment to all children with bone cysts who had suffered a pathologic fracture or the failure of earlier treatment. The treatment combination consisted of elastic intramedullary nailing (two ascending Titanium Nails, diameter depending on the medullar canal between 2.0 an 3.0 mm, Fa. Santech Nord, Germany), curettage, artificial bone substitute (Orthoss^®^, Fa. Geistlich, Germany) and autologous platelet rich plasma (GPS^®^, Biomet Merck Biomaterials, Berlin, Germany). Orthoss^® ^is an inorganic bone matrix with a macro- and microporous structure derived from bovine material. With the interconnecting pore structure and high inner surface it is an osteoconductive matrix, which is structurally integrated into the surrounding bone and incorporated into the physiological remodeling process. It is indicated for the filling of bone voids following trauma, for reconstruction in orthopedics and in spinal surgery [26-28]. The material has been in use for more than 20 years and more than 4 million applications are documented [29]. This new treatment combination was carried out with the informed consent of the parents and the patients themselves.

For each subject in the study, the following data are presented: age, gender, location and histology of cyst, earlier treatment history, peri- and postoperative morbidity and further operative procedures. Prior to operation, the distal-proximal, medial-lateral and anterior-posterior extent of every cyst was digitally determined from plain X-ray images.

Patients taking medicines known to influence platelet function or patients who had a platelet count < 100/nl were excluded from the study.

**Figure 1:**
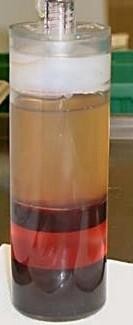
**GPS^®^-Tube after centrifugation**. The GPS^® ^tube after centrifugation: The red blood cells fraction at the bottom of the tube is separated from the buffy coat by a buoy. The platelet-rich plasma is above and is separated from the platelet-poor plasma after the white plunger is manually pushed down.

The autologous platelet rich plasma was augmented by the commercially available GPS^®^-System [30,31]. A blood sample of 40 to 110 ml was taken from each of the patients during anesthesia. The volume taken depended on the size of the cyst and the age of the patient. The preparation of GPS^® ^was performed in the operating theatre during the actual surgical intervention and took 20 minutes. In this procedure the blood was separated into three basic components: red blood cells, platelet poor plasma and 10-20 ml platelet-rich plasma (Figure 1).

Surgery was always performed under general anesthesia. After reduction of the fracture the elastic intramedullary nailing (ESIN) was performed under fluoroscopic guidance in an ascending matter (2-C-configuration). The diameter of the nails (2.0 to 3.0 mm) was selected on the basis of the preoperative anterior-posterior radiograph (digitally measured). In the cases of failed earlier treatment, elastic intramedullary nails were removed first. The production of GPS did not require any lengthening of the operation or the time of anesthesia, as it was carried out simultaneously. In all cases the bone cysts were then opened in a minimally invasive manner by an approximately 2 cm long incision. A small specimen for histological investigation was taken and the cyst debrided. Afterwards the 10 to 20 ml GPS^® ^(platelet rich plasma) was mixed with the artificial bone substitute (Orthoss^®^) and the cyst filled up with this mixture as completely as possible. The treatment protocol involved no further immobilization following intracutaneous wound closure.

All patients were reviewed with clinical examination, X-rays and functional evaluation four weeks after the operation, then every three months until complete bone mineralization occurred and removal of the nails was possible. Because long-term results were unknown we arranged one further visit one year after the nails were removed.

Treatment results were classified according to the scheme used by Capanna:

- Grade 1 = healed - the cyst was completely filled in with bone and the cortical margin thickened

- Grade 2 = healed with residual cyst - the cyst was consolidated with bone and the cortical margin thickened but there were still residual cyst parts

- Grade 3 = recurrence - the cyst initially consolidated with bone, but large areas of osteolysis and cortical thinning subsequently recurred

- Grade 4 = no response - the cyst showed no evidence of response to the treatment [4].

Grades 1 and 2 were defined as success, whereas grades 3 and 4 represented a failure in treatment. Statistical analysis was descriptive: averages and ranges were determined. Because the expected number of subjects was below twenty, no statistical tests were performed.

### Ethics

The study confirmed to the Helsinki Declaration and was approved by the local ethics committee of the University of Luebeck [AZ 10-223].

## Results

### Study participations

A cohort of twelve children (four girls, eight boys) was recruited. Mean patient age was 11.4 years (range 7-15 years) at the time of surgery. Histologically the bone defects included eight juvenile and four aneurysmal bone cysts (Table 1).

**Table 1:** Clinical characteristics of the study population

	Age	Sex	Cyst type *	Cyst Location	Cyst Size (mm)**	Acute Fracture	Prior treatment and Complication	Time to nail removal (month)	Follow-up (month)	Outcome Grade (Capanna)
1	10.4	f	JBC	Humerus proximal	47 × 12 × 14	+	-	7	41	2

2	11.8	f	ABC	Humerus proximal	39 × 19 × 15	-	ESIN + (Cerasorb^®^) for 3.5 years, nail exchange twice, Valgus, Capanna 4	7	39	1

3	13.3	m	JBC	Humerus central	75 × 23 × 17	-	ESIN alone, 2.5 years, Capanna 4	12***	37	2

4	15.5	m	ABC	Femur distal	65 × 22 × 14	+	-	6	36	1

5	11.0	m	JBC	Humerus proximal	66 × 13 × 22	-	ESIN alone > 4 years, nail exchange three times, Capanna 3	12	34	1

6	9.9	m	JBC	Humerus central	57 × 20 × 19	+	-	5	32	1

7	12.5	f	JBC	Humerus proximal	40 × 27 × 15	+	-	11	31	1

8	15.3	f	ABC	Humerus proximal	50 × 23 × 23	-	ESIN alone, > 2.5 years, Capanna 4	7	30	1

9	14.0	m	ABC	Humerus proximal	62 × 24 × 20	+	-	8	28	1

10	9.3	m	JBC	Femur proximal	36 × 29 × 18	+	-	8	28	1

11	9.7	m	JBC	Humerus proximal	55 × 19 × 18	-	ESIN alone > 3 years, nail exchange once, Capanna 3	4.5	25	1

12	7.0	m	JBC	Humerus proximal	48 × 18 × 19	+	-	5	20	1

* JBC = Juvenile bone cyst; ABC = Aneurysmal bone cyst

** To estimate the size of the defects, every cyst was digitally measured from the plain X-rays in mm prior to operation: distal-proximal, medial-lateral and anterior-posterior.

*** One 14 year old boy wished a further GPS^® ^and Orthoss^® ^application at the age of 14 years because of a small residual cyst (6 ml) and complicated prior treatment with ESIN alone.

**Figure 2:**
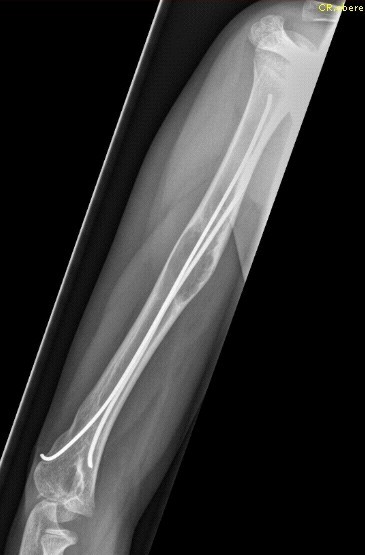
**Failed Consolidation after treatment with nailing alone**. Persistence of cyst after earlier failed treatment (Elastic stable intramedullary Nailing 4 years previously)

**Figure 3:**
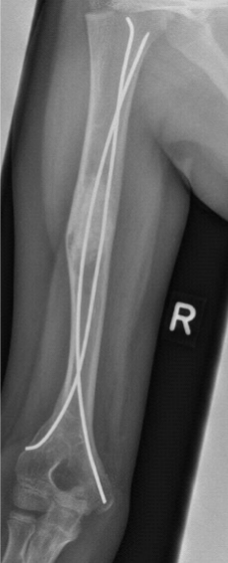
**Failed Consolidation after treatment with nailing alone**. Elastic stable intramedullary Nailing in combination with Orthoss^® ^and GPS^® ^after earlier failed treatment. Radiograph three months following the initial GPS^®^/Orthoss^® ^treatment resulting in bone mineralization.

**Figure 4:**
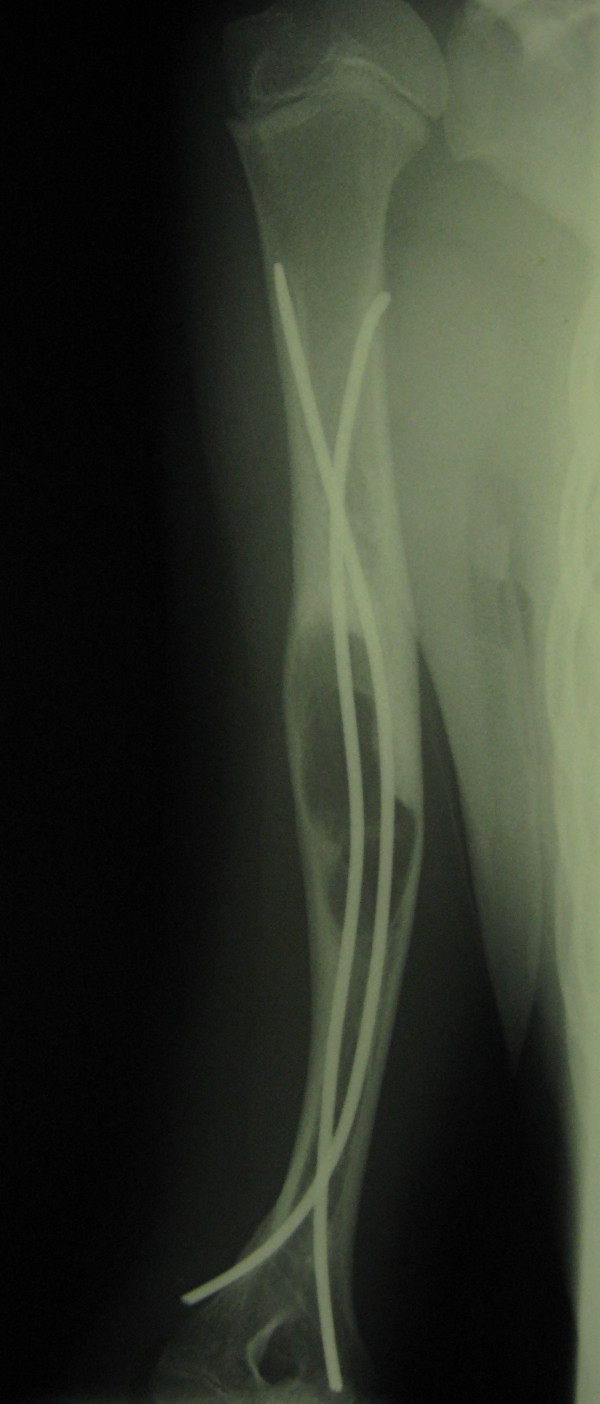
**Complete Consolidation after a long history of failed treatment**. Valgus deformity of the humerus after failed earlier treatment of juvenile bone cyst with Elastic stable intramedullary Nails and a different artificial bone substitute. After 3 years of failed treatment the result was classified as Capanna Grade 4. During removal of the nails and the combined treatment with ESIN-osteosynthesis, Orthoss^® ^and GPS^® ^an additional external fixation was needed due to instability. The Fixateur was removed after 4 weeks, the nails after 6 months.

**Figure 5:**
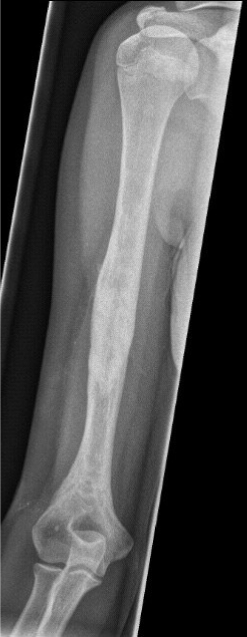
**Complete Consolidation after a long history of failed treatment**. Results 13 months after ESIN and treatment with Orthoss^® ^and GPS^®^. Clinically the patient achieved an excellent functional result without refracture or deviation of axis.

**Figure 6:**
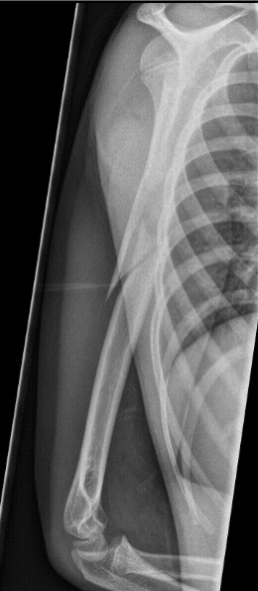
**Patient presenting with acute pathologic fracture.** Consolidation with ESIN, Orthoss^® ^and GPS^®^. Pathologic fracture of the right humerus after a fall on wet grass.

**Figure 7:**
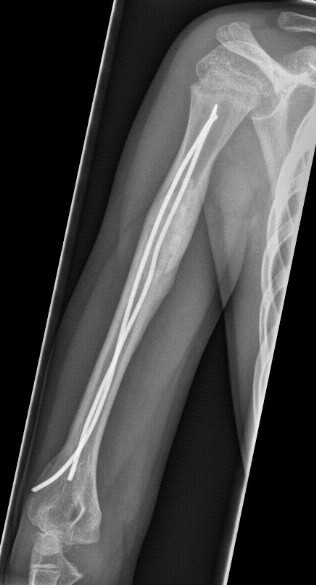
**Patient presenting with acute pathologic fracture.** Consolidation with ESIN, Orthoss^® ^and GPS^®^. Lateral view X-ray five months after treatment with ESIN, Orthoss^® ^and GPS^®^. As a consequence, Nails could be removed.

**Figure 8:**
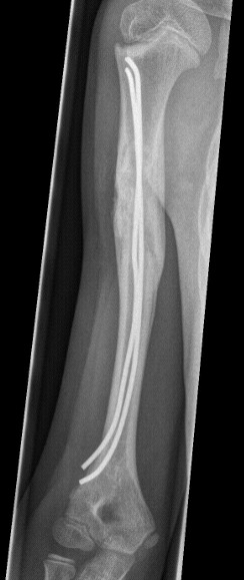
**Patient presenting with acute pathologic fracture.** Consolidation with ESIN, Orthoss^® ^and GPS^®^. Anterior-posterior X-ray five months after treatment with ESIN, Orthoss^® ^and GPS^®^. As a consequence, Nails could be removed.

**Figure 9:**
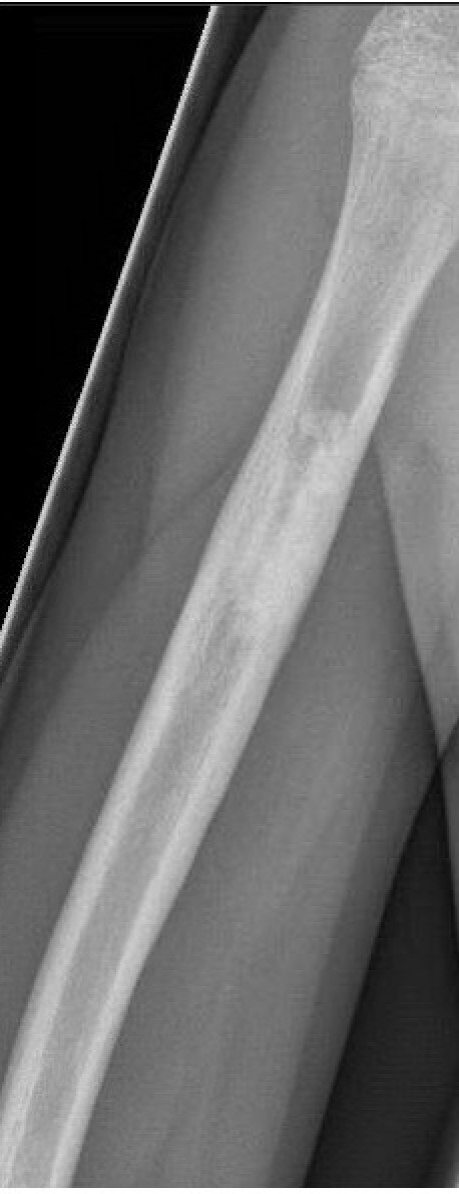
**Patient presenting with acute pathologic fracture.** Consolidation with ESIN, Orthoss^® ^and GPS^®^. 12 months after nail removal. Capanna Typ 1.

Five patients had suffered prior unsuccessful treatment of humeral bone cyst after pathologic fracture with intramedullary nailing and curettage (Figures 2, 3, 4 and 5) or artificial bone substitution in one case; the other seven presented with acute pathologic fractures (five humeral, two femoral; Figures 6, 7, 8 and 9). They all received the treatment combination of elastic intramedullary nailing (ESIN), artificial bone substitute (Orthoss^®^) and autologous platelet rich plasma (GPS^®^). None satisfied the exclusion criteria.

Two further patients (one boy, one girl) refused to participate. The pathologic fractures of these patients were stabilized with ESIN alone. Another patient excluded from this study presented with a 30 mm × 40 mm × 50 mm sized cyst of the calcaneus. After debridement, the defect was filled with Orthoss^® ^and GPS^®^. X-rays after 3 and 6 months showed complete mineralization of the former cyst.

### Peri-and postoperative morbidity

No side effects were obvious before and after the admission of ESIN, Orthoss^® ^and GPS^®^. No postoperative complications such as infection, deviation of axis, re-operation of intramedullary nailing or refractures occurred.

### Follow up

At the first outpatient visit, four weeks after the operation, all patients reported complete pain relief from an average time of one week after the surgery. The time taken for the patients to return to full, unrestricted activities was four to six weeks. The radiological findings at four weeks showed the beginning of fracture healing and mineralization of the defect. After three months no deviation of axis was evident and radiological examination revealed that the cysts had begun to heal.

**Figure 10:**
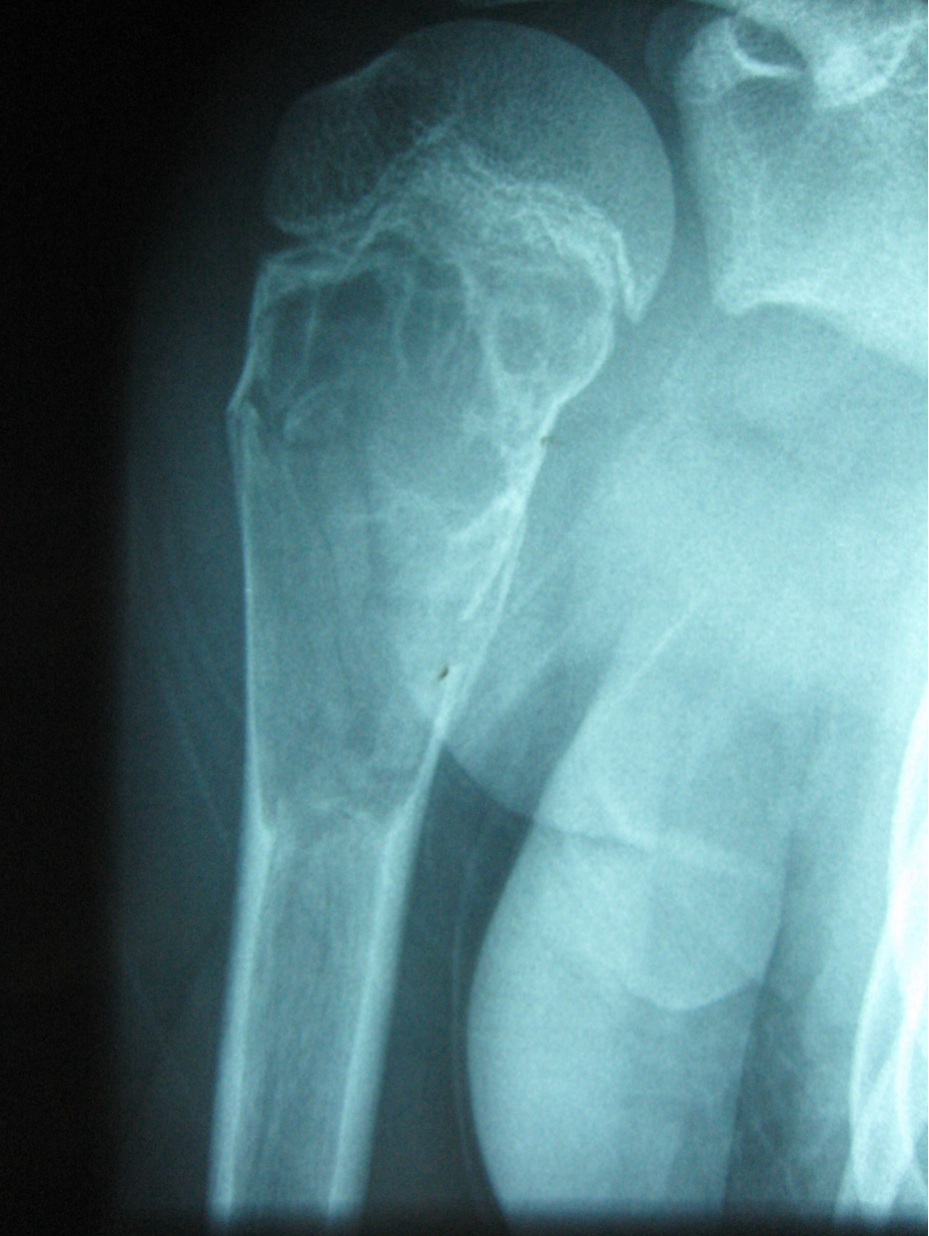
**Incomplete filling of the cyst**. Acute pathologic fracture of the right proximal humerus.

**Figure 11:**
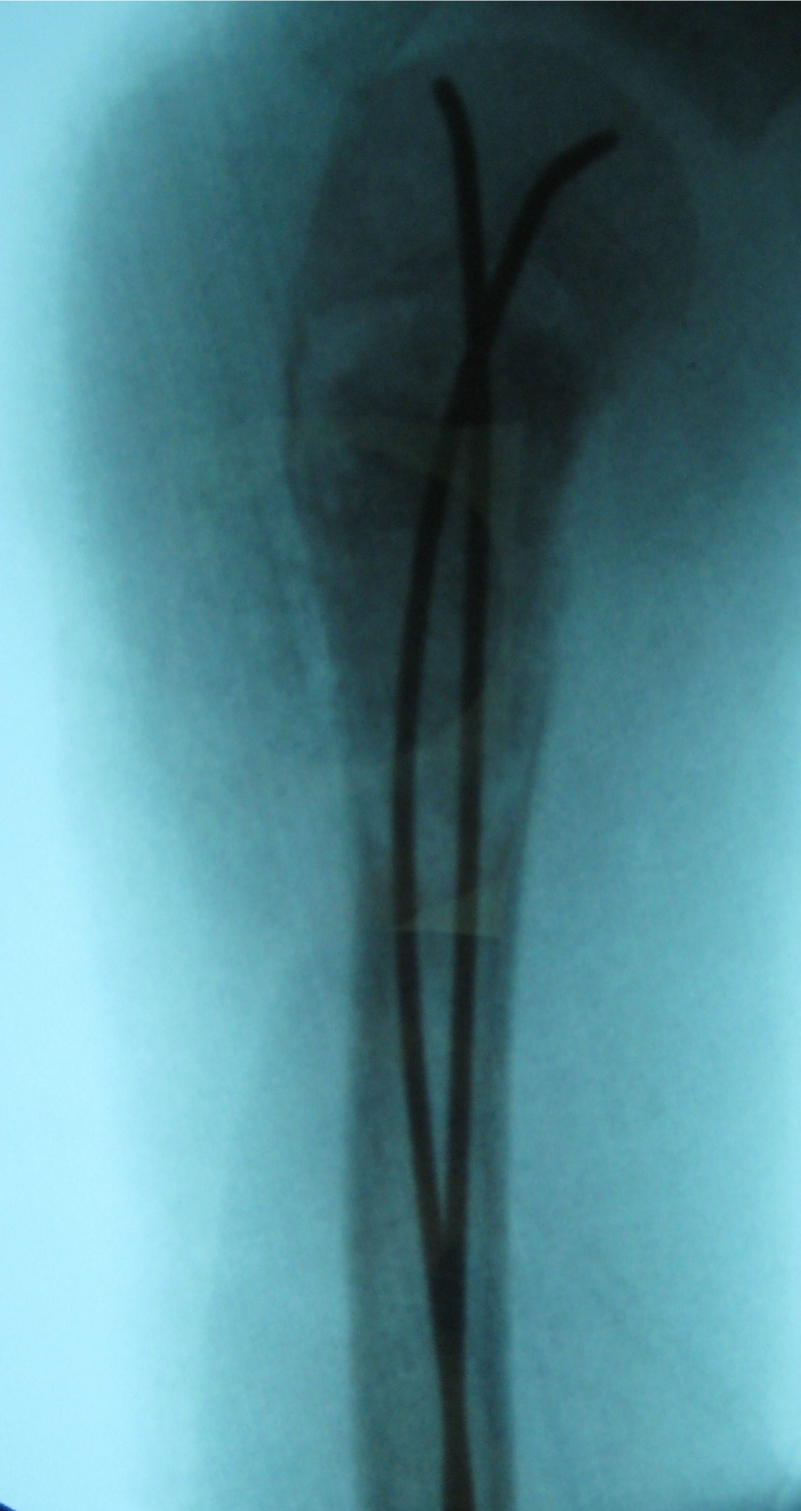
**Incomplete filling of the cyst**. Intraoperative fluoroscopic control after Orthoss^® ^and GPS^® ^application. Small filling defect in the upper part of the former cyst visible.

**Figure 12:**
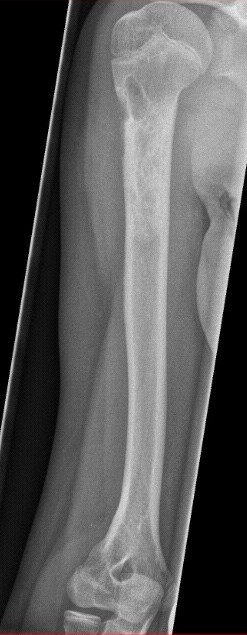
**Incomplete filling of the cyst**. 23 months after nail removal. Small residual cyst in the area of the former postoperative defect (Capanna Typ 2).

The radiological findings at six month showed a total resolution of the cysts in ten cases (Capanna Grade 1); in two cases a small residual cyst remained (Capanna Grade 2). All fractures healed and complete bone mineralization had occurred. The tiny residual cysts remained on the proximal end of the cyst. The patients with and without a residual cyst had identical treatment strategy. In the two cases with residual cysts, the intraoperative X-rays clearly revealed a technical error: an incomplete filling of these parts of the cysts (Figures 10, 11 and 12). The intramedullary nailing was removed five to twelve months after the operation (mean 7.7 months); in one case a fourteen year old boy (Capanna Grade 2) wished a further GPS^® ^and Orthoss^® ^application to reach a total resolution. All patients are still being followed up (20-41 months, mean 31.8 months), they show good functional results without any movement limitations and no refracture occurred after they returned to sports.

## Discussion

The question as to whether a conservative or operative treatment is the best treatment strategy in unicameral bone cysts, has been an active one since the Neer's observational series of about 175 cases. Children and adolescents with bone cysts often present first with pathologic fractures. While gross dislocation calls for reduction and stabilization, immobilization can be the treatment of choice for cases with little dislocation or in cysts detected by chance. However, one or more refractures are known to occur in around ten percent of all children [16]. These complications are the reason why children limit their normal physical activities, which inhibits their social development. In the longer term, all lesions in younger children show high persistence and recurrence rates following conservative treatment. In the proximal humerus and femur - the most frequent localizations - the prognosis following both treatment options was reported to be less satisfactory. As a consequence, NEER supported prompt surgical intervention with curettage and bone grafting as "the best way to rehabilitate these young children" producing successful healing in 55% to 65% of cases [32].

Other treatment strategies such as filling the cysts with cortisone [3,5,6], bone marrow [7-9,33,34] or allogenic bone grafts were evaluated [10-12,35]. Long-term studies of percutaneous injection of methylprednisolone acetate have not supported the initial satisfactory results with success rates of only about 50 to 60% [36]. Failure rates of about 80% for cortisone, 64% for curettage and 50% of combined procedures with bone marrow have been reported [16]. In a case series Lokiec reported the consolidation of bone cysts in all ten patients treated with percutaneous autologous marrow grafting allied to multiple perforations of the cysts before injection [33], which - in our opinion - will additionally weaken the affected bone. All these described methods may produce consolidation of the cyst but they do not initially enhance the mechanical stability of the weakened bone [37]. As a consequence patients have to avoid strenuous activities and sports for the duration of the healing process, which might be years [6,16]. In our experience, this is not seen as a valid therapeutic option by parents and patients.

Mechanical stability was achieved by the improvement in pediatric surgery with elastic intramedullary nailing of the pathologic fractures. It is described as a minimal invasive surgery, which supports the healing of the cysts. Stabilization of the bone allows early mobilization of the patients without major complications [14,37]. The disadvantage is the moderate success rates of about 70% for complete healing and the prolonged healing period, necessitating the exchange of the elastic stable nails once or twice during therapy [14]. In two of our patients the removal of the nails was really difficult and one further patient experienced an unsuccessful attempt to remove the nails in another hospital. The difficult changing operations and concomitant prolonged periods of postoperative immobilization render this method unconvincing [13,38]. In our study earlier treatment with ESIN on its own had failed in four patients, which was also the case in another cyst that was additionally treated with a product containing tricalciumphosphate (Cerasorb^®^). The duration of their treatment history was four years and two of the children underwent multiple operations.

The combined biological and mechanical treatment of simple bone cysts was described for the first time by Kanellopoulos. His treatment employed demineralized bone matrix and autologous bone marrow injection in addition to intramedullary nailing for the stabilization of bone cysts. In seven patients the cysts consolidated completely; two consolidated partially [13,38]. Although autologous bone marrow collection is considered a relatively simple procedure, it can be associated with numerous complications such as biopsy site bleeding, hematoma or infection [39,40]. Harvesting enough bone marrow for huge cysts in children is sometimes problematic and causes more pain than filling the cyst and implanting the nails. To avoid these complications, we added GPS^® ^and Orthoss^® ^to our treatment, the rationale being that the application of growth factors from the platelet-rich plasma has been shown to promote tissue repair in many other clinical situations such as cranio-facial surgery [41].

With the combined mechanical and biological treatment of elastic intramedullary nailing, artificial bone substitute and autologous platelet rich plasma, we describe an option having the possibility of early removal of the intramedullary nailing (six to twelve months, mean 7.7 month), thus avoiding changing operations. In our patients it prevented further pathologic fractures and was able to avoid long-lasting limitations in activity. Our approach lead to visible (2 - 3 cm) scars on the upper arm or the leg and two further small incisions for the nails, but the children were satisfied with their appearance. No patient showed any changes in their intra- or postoperative vital parameters. The most significant benefits of using the GPS^®^-System are its autologous nature, the fact that it is endogenously derived and its easy availability. There are no issues of immunogenicity or transmission of infection by using the GPS^®^-System or the artificial bone substitute Orthoss^®^. Hass reported the successful use of demineralized human bone matrix (Grafton^®^) for the treatment of bone cysts in seven children. The disadvantages of this approach are the high costs and the, albeit low, risk of infection [42].

Another interesting study from 2010 published data on 24 patients treated with ChronOS^® ^(Synthes, Switzerland). Treatment with this new synthetic tricalciumphosphate cement resulted in successful healing of different bony lesions in 19 cases; in two others healing with residual cyst. During follow-up from one to twenty months ten defects were observed with partial or subtotal absorption of the injected cement. The volume injected ranged from 2-30 ml [43].

In our series the radiographic evaluation showed complete healing in 10 of 12 patients; only two patients had a small residual cyst in the upper part of the earlier cyst. Apart from one patient, no X-rays were performed later than three years after removal of the implant, because cysts are known not to recur once totally consolidated after treatment [3]. Rougraff demonstrated, that most cyst recurrence occurred at their proximal or distal ends and suggested that this might be related to incomplete filling of the end of the cysts [11]. Retrospective analysis of all X-rays from the intraoperative fluoroscopic films confirmed that the filling of the upper part of the cyst was incomplete in our 2 cases with small residual cysts.

In all patients the removal of the nails was possible after only six to twelve months (mean 7.7 month); such short treatment period has never been previously reported in the literature. Rougraff showed radiographically, that by six to nine months, mature cortical thickening was present in most of his patients, and it was seen in almost every patient by one year. He confirmed that the radiographic features changed very little after one year following treatment [11]. From our data we can conclude that the combination with Orthoss^® ^and GPS^® ^will fasten the healing of the cysts.

Two years after the combined treatment strategy with elastic intramedullary nailing (ESIN), artificial bone substitute (Orthoss^®^) and autologous platelet rich plasma (GPS^®^) our preliminary results are promising, but further prospective studies are necessary to validate the efficacy. Although this is a small series that lacks a control group, the results were excellent with no mayor complications such as re-fracture or re-operation (apart from possible early implant removal). The time to healing was short and the children returned early to full daily activities, having suffered restrictions for no more than one month. At the latest examination more than one year after implant removal there was no clinical evidence of cyst recurrence and the functional results were optimal.

In our opinion, our promising results as well as the results of the treatment with Grafton^® ^[42] and ChronOS^® ^[43] justify a prospective multicenter study for further evaluation.

## Conclusions

The combination of elastic intramedullary nailing, artificial bone substitute (Orthoss^®^) and autologous platelet rich plasma (GPS^®^-System) enhances the treatment of bone cysts in children. It is a safe method without additional perioperative complications and it shortened total treatment time in our series compared to earlier strategies. Secondary procedures such as difficult removal of the elastic stable intramedullary nails followed by a new implantation as well as re-do surgery due to refractures or deviation of axis were avoided. Technically the decisive factor is the debridement of the cyst with the complete filling of the cyst with artificial bone substitute and autologous platelet rich plasma to avoid residual cysts.

## Competing interests

The authors declare that they have no competing interests.

## Authors' contributions

LMW and MMK participated in the planning of the study and did the operations. MR and DS had responsibility for data collection and participated in writing the paper. MMK coordinated the study and had overall responsibility. LMW and MMK revised the manuscript critically for important intellectual content. All authors (MR, DS, LMW and MMK) read and approved the final manuscript.

## Pre-publication history

The pre-publication history for this paper can be accessed here:

http://www.biomedcentral.com/1471-2474/12/45/prepub
